# Association between Thyroid Function and Insulin Resistance Indices in Korean Adolescents: Findings from the 2014–2015 Korea National Health and Nutrition Examination Survey

**DOI:** 10.3390/children11030370

**Published:** 2024-03-20

**Authors:** Eunji Mun, Hye Ah Lee, Jung Eun Choi, Rosie Lee, Kyung Hee Kim, Hyesook Park, Hae Soon Kim

**Affiliations:** 1 Department of Pediatrics, College of Medicine, Ewha Womans University, Seoul 07804, Republic of Korea; silverji16@naver.com (E.M.); cjeped@ewha.ac.kr (J.E.C.); dlfhwl@naver.com (R.L.); gonato37@naver.com (K.H.K.); 2 Clinical Trial Center, Ewha Womans University Mokdong Hospital, Seoul 07985, Republic of Korea; khyeah@ewha.ac.kr; 3 Graduate Program in System Health Science and Engineering, Department of Preventive Medicine, College of Medicine, Ewha Womans University, Seoul 07804, Republic of Korea; hpark@ewha.ac.kr

**Keywords:** thyroid function, insulin resistance, adolescent, Korea National Health and Nutrition Examination Survey (KNHANES)

## Abstract

Aim: This study investigated the sex-specific association between thyroid function and various insulin resistance (IR) indices, including noninsulin-based IR indices, in euthyroid adolescents. Methods: A total of 465 adolescents (aged 12–18 years; 255 boys and 210 girls) based on data from the 2014–2015 Korea National Health and Nutrition Examination Survey were included. Serum thyrotropin (thyroid-stimulating hormone [TSH]) and free thyroxine (fT4) were used to assess thyroid function, whereas the homeostasis model assessment of insulin resistance (HOMA-IR), quantitative insulin-sensitivity check index (QUICKI), glucose/insulin ratio (GIR), triglyceride–glucose (TyG) index, and triglyceride/high-density lipoprotein cholesterol (TG/HDL-C) ratio were used to assess IR. The relationship between thyroid function and IR was analyzed using multiple linear regressions stratified by sex, considering obesity status. Results: The relationship between thyroid function and IR varied depending on sex and was more pronounced in the overweight/obesity subgroup for both boys and girls. In overweight and obese boys and girls, fT4 was significantly associated with HOMA-IR and QUICKI with conflicting association directions. TSH was also positively associated with the TyG index in both sexes. Conclusions: The findings suggest that the relationship between thyroid function and IR in adolescents might vary depending on sex, and the degree of association was significant in obese adolescents.

## 1. Introduction

Insulin resistance (IR) is characterized by the diminished responsiveness of cells to the glucose-lowering effects of both exogenous and endogenous insulin compared to the normal population [1]. IR is a risk factor for hyperglycemia and dyslipidemia, which are associated with metabolic syndrome [2,3,4,5]. Moreover, measuring IR is crucial as metabolic syndrome is strongly linked to cardiovascular diseases [2,3,4,5]. The hyperinsulinemic euglycemic clamp method directly measures IR [3,4]. However, its feasibility is limited in typical clinical and research settings [3]. Therefore, IR has been primarily evaluated using indices such as the homeostasis model assessment of insulin resistance (HOMA-IR), quantitative insulin-sensitivity check index (QUICKI), 1/fasting insulin, and glucose/insulin ratio (GIR) [3,4]. However, these methods require measuring insulin to assess IR, considering the issues with storing and the cost of fasting insulin measurements [5]. Consequently, the triglyceride–glucose (TyG) index and triglyceride/high-density lipoprotein cholesterol (TG/HDL-C) ratio were developed as noninsulin-based IR indices [5,6].

Thyroid hormones play a vital role in maintaining energy balance and regulating diverse metabolic processes, including glucose and lipid metabolism [2,6]. Hypothyroidism is related to type 2 diabetes mellitus (T2DM) and metabolic syndrome [6]. Elevated thyroid function disrupts glucose metabolism in peripheral tissues and affects hepatic glucose metabolism, resulting in IR [7]. Thyroid hormones directly and indirectly influence glucose metabolism, particularly by promoting glucose production in peripheral tissues, such as fat and muscles, thereby increasing IR [7].

Many studies have investigated the relationship between thyroid function and IR [8,9,10,11,12]. However, research focusing on adolescents and evaluating various IR indices is limited. Previous studies mostly assessed IR using HOMA-IR or QUICKI. Meanwhile, some studies investigated the relationship between TyG index and thyroid-stimulating hormone (TSH) and free thyroxine (fT4) levels in adults with normal thyroid function, showing a significant association [6,13]. Additionally, one study suggested that the TyG index might provide a more reliable assessment of IR concerning thyroid function compared with HOMA-IR [6].

This study aimed to investigate sex-specific associations between thyroid function and various IR indices, including noninsulin-based IR indices, in euthyroid adolescents using data from the 2014–2015 Korea National Health and Nutrition Examination Survey (KNHANES). We also assessed whether this association remained across subjects with and without overweight and obesity.

## 2. Methods

### 2.1. Data Source and Study Subjects

This study analyzed data from the 2014–2015 KNHANES, a survey data source representing the Korean population. Each year, the Korea Centers for Disease Control and Prevention administers KNHANES, a population-based cross-sectional study consisting of three parts: health interview, health examination, and nutritional survey. The health examination involves collecting data on various health-related indicators, as well as blood (after fasting) and urine sampling for a biochemical profile and anthropometric measurements. More information on KNHANES can be found elsewhere [14].

We included subjects aged 12–18 years from the KNHANES dataset, with 7550 individuals in 2014 and 7380 in 2015 (*n* = 1134; 551 in 2014, 583 in 2015). Exclusion criteria comprised subjects with missing TSH, fT4, and fasting blood glucose (FBG) data, as well as those with DM (*n* = 615). Subjects whose thyroid function was abnormal (0.59 ≤ TSH ≤ 7.03 and 0.92 ≤ fT4 ≤ 1.6) [15] or whose thyroid peroxidase antibody (TPO Ab) was positive were also excluded (*n* = 54). Ultimately, 465 subjects (255 boys and 210 girls) were included for analysis.

The open KNHANES data did not contain personal information, and ethical approval for the use of open KNHANES data was exempted by the Institutional Review Board Committee of Ewha Womans University Hospital (IRB no. 2024-01-016).

### 2.2. Measurements

Height and weight were measured to the nearest 0.1 cm and 0.1 kg, respectively, while not wearing shoes and wearing light indoor clothes. Body mass index (BMI) was calculated by dividing weight in kilograms by the square of height in meters. Subjects with a BMI ≥ 85th percentile for age and sex based on the 2017 Korean National Growth Charts for children and adolescents were defined as overweight [16]. Meanwhile, subjects with a BMI ≥ 95th percentile for adolescents of the same age and sex were defined as obese.

The collected specimens were subjected to laboratory tests at a certified institution within 24 h. FBG, TG, and high-density lipoprotein cholesterol (HDL-C) levels were measured enzymatically using the Hitachi 7600-210 Automatic Analyzer (Hitachi, Tokyo, Japan).

Serum insulin concentrations were measured using a gamma counter with an immunoradiometric assay (INS-Irma, Biosource, Nivelles, Belgium). However, the concentrations were measured only in 2015. Thyroid function was assessed using serum TSH and fT4 levels measured by electrochemiluminescence immunoassays (Roche Diagnostics, Mannheim, Germany). TPO Ab was measured using an E-anti-thyroid peroxidase kit by electrochemiluminescence immunoassays (Roche Diagnostics, Mannheim, Germany).

### 2.3. Insulin Resistance Indices

HOMA-IR, QUICKI, GIR, TyG index, and TG/HDL-C ratio were included as IR indicators. HOMI-IR was calculated by the following equation: fasting insulin (µIU/mL)× FBG (mg/dL)/22.5. QUICKI was obtained by using the following equation: 1/(log[FBG (mg/dL)]+ log[fasting insulin (µIU/mL)]). The GIR was calculated by dividing FBG (mg/dL) by fasting insulin (µIU/mL). The TyG index was calculated as log (fasting TG [mg/dL]× FBG [mg/dL])/2. Finally, the TG/HDL-C ratio was calculated by dividing fasting TG (mg/dL) by HDL-C (mg/dL) [3,4,5,17]. Based on data availability, HOMA-IR, QUICKI, and GIR were evaluated only for 2015 data.

### 2.4. Covariates

Demographic factors, BMI, physical activity, and parental history of DM were considered potential covariates. Household income was expressed in quartiles (low, medium–low, medium–high, or high), defined as monthly household income divided by the square root of household size. Physical activity data from the KNHANES were collected using the International Physical Activity Questionnaire. Aerobic exercise was defined as at least 150 min of moderate physical activity per week, at least 75 min of vigorous physical activity per week, or an equivalent mixture of moderate and vigorous physical activity (where 1 min of vigorous activity is equivalent to 2 min of moderate activity). Muscle-strengthening exercise was defined as practicing strength training at least 2 days a week [18,19]. Additionally, parental history of DM was defined through a questionnaire if the father or mother was diagnosed with diabetes by a doctor.

### 2.5. Statistical Analysis

All statistical analyses were conducted using SAS version 9.4 (SAS Institute Inc., Cary, NC, USA). The analyses were performed using the PROC SURVEY procedure considering the complex sampling design of KNHANES. Descriptive statistics were presented as weighted means with 95% confidence intervals (95% CIs) for numeric data and unweighted frequencies with weighted percentages for nominal data. Nonnormally distributed numerical data were log-transformed. For basic characteristics, SURVEYREG and SURVEYFREQ were used to assess sex differences. Linear regression analysis was used to assess the linear associations between thyroid function and IR indicators, and the results were expressed as beta coefficients and 95% CIs. Additionally, given the relatively small population of overweight and obese subjects, analyses were stratified by normal weight, overweight, or obesity to increase the precision of the results.

The P for the interaction between IR indicators and obesity status was estimated by adding an interaction term to the statistical model. Sex, age, household income (quartiles, continuous), BMI, muscle exercise (yes/no), and parental history of DM (yes/no) were included as covariates. Linear regression analysis was used to assess the association of covariates with TSH and fT4 levels. Additionally, nonlinearity between thyroid function and IR indicators was assessed through restricted cubic splines with knots at the 10th, 50th, and 90th percentiles by adjusting for covariates. It was obtained by applying individual survey weights. *p* < 0.05 indicated statistical significance under two-tailed tests.

## 3. Results

### 3.1. Baseline Characteristics of Study Participants

Table 1 presents the clinical characteristics of the subjects. The average age of boys and girls was 15.18 and 14.95 years, respectively (*p* = 0.253). Compared with girls, boys had a higher BMI (22.32 kg/m^2^ vs. 21.45 kg/m^2^, *p* = 0.028).

TSH (2.86 uIU/mL vs. 2.52 uIU/mL, *p* = 0.013), fT4 (1.33 ng/dL vs. 1.24 ng/dL, *p* < 0.001), and FBG (92.9 mg/dL vs. 90 mg/dL, *p* < 0.001) levels were higher in boys than in girls. No sex differences regarding serum insulin level or any of the IR indicators were observed. Among basic characteristics, age was positively associated with fT4 levels in boys (*β* = 0.016, 95% CI 0.006–0.026, *p* = 0.002) (Supplementary Table S1).

### 3.2. Association between Thyroid Function and IR Indices

By analyzing the sexes separately, a positive association was observed between fT4 levels and QUICKI (*β* = 1.66, *p* = 0.047) and GIR among boys (*β* = 0.04, *p* = 0.049). However, the relationship became nonsignificant after adjustments. Among girls, fT4 showed a significant negative association with HOMA-IR (*β* = −0.05, *p* = 0.045) and a significant positive association with GIR (*β* = 0.06, *p* = 0.022) after adjustments (Table 2).

### 3.3. Nonlinearity Assessment between Thyroid Function Test and IR Indices by Sex

**Figure 1:**
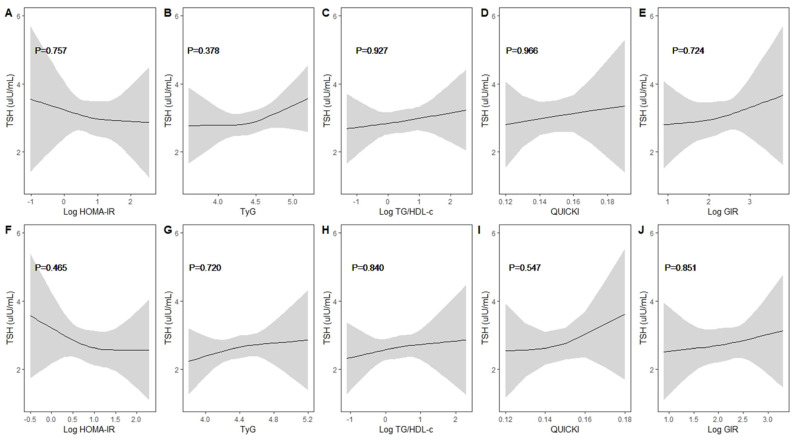
Non-linearity assessment between thyrotropin and insulin resistance indices by sex. (**A**–**E**): boys and (**F**–**J**): girls. Results were obtained through restricted cubic splines with knots at the 10th, 50th, and 90th percentiles by adjusting for age, household income (quartiles, continuous), BMI, muscle exercise (yes/no), and parental history of DM (yes/no), with individual survey weights allowed. The solid line represents the adjusted means, and the shaded area indicates the 95% confidence intervals. None of the insulin resistance indices showed non-linearity with thyrotropin.

**Figure 2:**
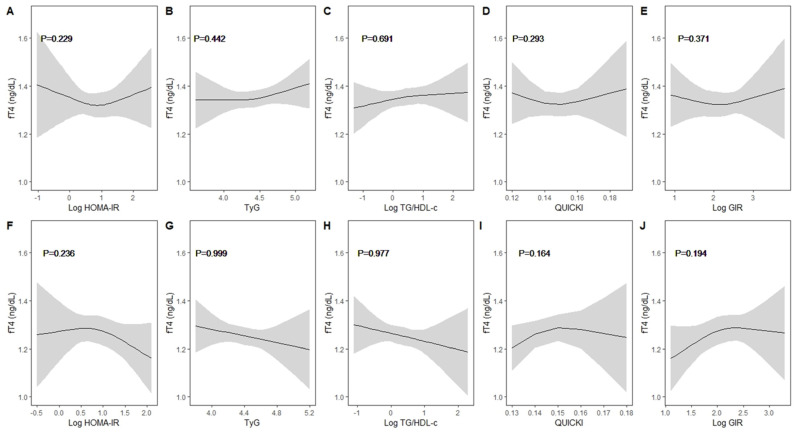
Non-linearity assessment between free thyroxine and insulin resistance indices by sex. (**A**–**E**): boys and (**F**–**J**): girls. Results were obtained through restricted cubic splines with knots at the 10th, 50th, and 90th percentiles by adjusting for age, household income (quartiles, continuous), BMI, muscle exercise (yes/no), and parental history of DM (yes/no), with individual survey weights allowed. The solid line represents the adjusted means, and the shaded area indicates the 95% confidence intervals. None of the insulin resistance indices showed non-linearity with free thyroxine.

A nonlinearity assessment of thyroid function and IR indices stratified by sex was performed (Figure 1 and Figure 2). In boys, a linear trend was observed between thyroid function and IR indices. Meanwhile, a negative trend was observed between TSH and HOMA-IR, whereas the other IR indicators showed a positive trend with TSH. Likewise, girls exhibited a similar trend with linearity. FT4 also showed linearity and had different directions of influence in boys and girls.

### 3.4. Association between Thyroid Function and IR Indices in the Presence or Absence of Obesity

The subjects were divided into normal weight and overweight/obese groups. Then, the relationship between thyroid function and IR indicators was analyzed (Table 3). A positive association between HOMA-IR and fT4 (*β* = 0.08, *p* = 0.037) and a negative association between QUICKI and fT4 (*β* = −4.14, *p* = 0.034) were observed among boys in the overweight/obese group. The TyG index was also positively related to TSH (*β* = 1.01, *p* = 0.035). Among girls, negative associations were observed between HOMA-IR and fT4 (*β* = −0.12, *p* = 0.002) and between TG/HDL-C and fT4 (*β* = −0.06, *p* = 0.046). Additionally, QUICKI (*β* = 6.60, *p* = 0.002) and GIR (*β* = 0.14, *p* = 0.001) were positively associated with fT4. Furthermore, the TyG index and TSH showed a positive relationship (*β* = 1.38, *p* = 0.011). In both sexes, the associations between fT4 and HOMA-IR and between fT4 and QUICKI differed significantly depending on obesity status (*p* < 0.05 for interaction).

## 4. Discussion

This study investigated the sex-specific associations between thyroid function and IR using various indices, including noninsulin-based IR indices, based on the 2014–2015 KNHANES data in Korean adolescents. In girls, a negative association between fT4 and HOMA-IR and a positive relationship between fT4 and GIR were observed. In the overweight/obese group, other IR indicators, including the TG/HDL-C ratio and QUICKI, were associated with fT4. In boys, no significant relationship was observed between thyroid function and IR indices. However, a positive association between HOMA-IR and fT4 and a negative association between QUICKI and fT4 were observed in the overweight/obese group. Furthermore, TSH exhibited a positive association with the TyG index in both sexes in the overweight/obese group.

Thyroid function is sex specific [20]. In a study conducted in Japan with a total of 2036 children and adolescents, boys generally had higher TSH and fT4 levels than girls at age ≥ 9 years [21]. Additionally, a study conducted in Australia found that adolescent boys had higher levels of TSH, fT3, and fT4 than girls [22]. These results are in line with our study. Some researchers explained that these differences are probably due to the effect of growth and sex hormones [21]. However, some studies found no sex differences in TSH and fT4 levels [23,24], indicating the need for further research on thyroid function and its association with sex in adolescents.

Thyroid hormones play a significant role in maintaining the balance of glucose levels in the body [8,25]. Ambrosi et al. suggested that exposure to organochlorine compounds, which accumulate in adipose tissue, might influence thyroid hormone synthesis in obese individuals [10]. Organochlorine-induced damage to thyroid follicles, which disrupts the production and release of T4, might explain the reduction in fT4 levels, accompanied by compensatory elevations in TSH, particularly in obese individuals with IR [10]. In a study of 303 individuals, increased subcutaneous fat accumulation in slightly overweight individuals with normal thyroid function was related to low fT4 and high TSH levels [26].

Moreover, the metabolically healthy normal weight group had higher fT4 levels compared with other groups according to obesity status and metabolic health in a recent cross-sectional study of 2988 euthyroid adults [27]. In obese individuals, a reduction in thyroid hormone receptors contributes to increased levels of TSH and peripheral thyroid hormones, disrupting the negative feedback mechanism between them [27,28]. Elevated leptin levels in adipose tissue further stimulate the hypothalamus–pituitary–thyroid axis, increasing thyrotropin levels [27]. Several studies revealed that obese children and adolescents have elevated TSH levels and reduced fT4 levels compared to their normal-weight counterparts [29,30,31]. A study using data from 1104 adolescents from KNHANES 2013–2015 found a higher prevalence of subclinical hypothyroidism among obese adolescents [32]. Therefore, it can be hypothesized that obesity-induced IR is associated with subclinical hypothyroidism. The association between thyroid function and IR is a topic warranting ongoing research in the future to further understand its underlying mechanisms and implications.

In our study, we observed differences in the directionality of the IR indices. As IR increased, both HOMA-IR and TyG index increased, while GIR and QUICKI decreased due to the increase in FBG levels [3,4,17]. Additionally, IR is associated with high TG and low HDL-C [33]. Therefore, as IR increases, there is a tendency for the TG/HDL-C ratio to increase [5]. Therefore, either positive or negative associations might exist depending on the indices used to evaluate the relationship between the IR indices and thyroid function within the same sex.

Research focusing on the relationship between thyroid function and IR in children and adolescents is limited. A retrospective longitudinal study conducted in Portugal investigated the relationship between obesity, thyroid function, and IR, and found that obese children with high TSH levels exhibited increased IR, with these positive correlations between TSH and HOMA-IR persisting even within the normal TSH range [8]. Additionally, a study involving euthyroid children revealed that high TSH levels and low fT4 levels are linked to increased IR indicators [9]. In studies involving adults, the direction of the relationship between thyroid function and IR varied across the indices. In a study of 581 euthyroid overweight and obese subjects in Italy, TSH was positively associated with HOMA-IR, whereas fT4 was negatively associated with HOMA-IR [10]. Conversely, the opposite results were observed with QUICKI [10]. These results are in line with those observed in overweight and obese girls in ours and another study [11]. In contrast, HOMA-IR was positively associated with fT4 levels in a study conducted on euthyroid individuals in the early T2DM stages [12]. We also observed these trends in overweight and obese boys. In the present study, an opposite-direction association was observed for the IR index between boys and girls, especially for fT4. In a study conducted in South Korea euthyroid adults, sex differences were observed in the relationship between TSH, fT4, and components of metabolic syndrome [34]. This might be explained by the influence of sex hormones on thyroid function and metabolic syndrome manifestation [34]. However, further research on the sex-specific association between thyroid function and IR in adolescents is needed to promote a greater understanding.

Studies demonstrated that the TyG index can serve as a valuable surrogate marker for detecting IR even in children and adolescents [35,36]. A cross-sectional study involving 345 children and adolescents classified as overweight and obese compared the predictive abilities of the TyG index and HOMA-IR for IR [35]. The findings indicated a positive correlation between the TyG index and HOMA-IR, demonstrating robust predictive capabilities for IR [35]. A study conducted on Korean children and adolescents also revealed the effectiveness of using the TyG index in predicting IR [36]. In a study investigating the relationship between the TyG index and TSH and fT4 levels in euthyroid adults from KNHANES 2015, the TyG index was positively related to TSH levels in women [6]. These findings are consistent with our results, especially regarding the relationship between the TyG index and TSH. Therefore, the TyG index might be beneficial in understanding the relationship between thyroid function and IR, even in adolescents.

Nevertheless, this study had some limitations. First, this study observed associations but could not establish causality between thyroid hormones and IR, considering its cross-sectional nature. Second, the limited scope of the study, including a single ethnicity, might have restricted its generalizability to more diverse ethnic groups. Third, thyroid hormone-related data were limited, comprising only TSH, fT4, and TPO Ab. Additionally, there were limitations in using fasting insulin data for all study subjects, possibly affecting the precision of the results for the association with thyroid function.

This study investigated sex-specific associations between thyroid function and IR among adolescents, particularly within the context of weight status, using a robust and nationally representative dataset. IR was evaluated using various IR indices, including the recently proposed TyG index. This is the first known study to evaluate thyroid function and IR, including the noninsulin-based IR indices, in adolescents. Furthermore, the relationship between thyroid function and IR indices might vary according to sex, with obesity further complicating this association. Emphasizing the importance of considering sex and obesity status when evaluating thyroid function is crucial. Our results will enhance the comprehension of the intricate relationship between thyroid hormones and IR in this population.

## 5. Conclusions

Thyroid function and IR in adolescents might differ depending on sex, and the degree of association was noticeable in obese adolescents. The TyG index showed promise in clarifying the relationship between thyroid function and IR in this age group. Although the clinical utility of TyG requires further exploration, understanding these associations can help clinicians assess metabolic health and develop interventions to reduce the risk of metabolic disorders in adolescents.

## Figures and Tables

**Table 1:** Basic characteristics and biochemical data of the study subjects.

	Total (n = 465)	Boys (n = 255, 54.89%)	Girls (n = 210, 45.11%)	*p*
Age (yrs)	15.08 (14.8–815.28)	15.18 (14.93–15.44)	14.95 (14.64–15.26)	0.253
Household income				
Q1 (low)	51 (11.50%)	22 (9.01%)	29 (14.57%)	0.367
Q2	122 (25.81%)	67 (26.17%)	55 (25.36%)	
Q3	166 (36.74%)	95 (38.68%)	71 (34.35%)	
Q4 (high)	122 (25.95%)	70 (26.13%)	52 (25.71%)	
Aerobic exercise	314 (70.08%)	187 (77.36%)	127 (61.50%)	0.001
Muscle exercise	121 (25.28%)	89 (34.97%)	32 (13.64%)	<0.001
Parental history of DM	28 (6.38%)	19 (8.26%)	9 (4.11%)	0.086
TSH (uIU/mL)	2.70 (2.57–2.84)	2.86 (2.68–3.03)	2.52 (2.32–2.72)	0.013
fT4 (ng/dL)	1.29 (1.28–1.31)	1.33 (1.31–1.35)	1.24 (1.22–1.26)	<0.001
Waist circumference (cm)	73.33 (72.29–74.36)	75.77 (74.39–77.15)	70.36 (68.96–71.75)	<0.001
Waist circumference (≥90th)	59 (13.97%)	31 (12.88%)	28 (15.30%)	0.540
BMI (kg/m^2^)	21.93 (21.52–22.34)	22.32 (21.78–22.87)	21.45 (20.88–22.03)	0.028
Normal weight	354 (75.20%)	192 (74.60%)	162 (75.93%)	0.954
Overweight (85–94th)	51 (10.30%)	29 (10.46%)	22 (10.11%)	
Obesity (≥95th)	60 (14.49%)	34 (14.93%)	26 (13.96%)	
FBG (mg/dL)	91.59 (90.91–92.28)	92.9 (92–93.81)	90 (88.98–91.03)	<0.001
Insulin (uIU/mL)	10.98 (10.21–11.81)	10.80 (9.79–11.91)	11.20 (10.02–12.52)	0.629
Total cholesterol (mg/dL)	158.63 (155.91–161.35)	154.46 (150.59–158.33)	163.71 (159.52–167.89)	0.003
TG (mg/dL)	73.46 (69.66–77.47)	74.70 (68.89–80.99)	71.98 (67.59–76.66)	0.475
HDL-C (mg/dL)	51.50 (50.45–52.54)	49.78 (48.39–51.18)	53.58 (52.12–55.05)	<0.001
Insulin resistance indices				
HOMA-IR	2.48 (2.29–2.67)	2.47 (2.23–2.73)	2.49 (2.21–2.79)	0.923
QUICKI	0.15 (0.14–0.15)	0.15 (0.14–0.15)	0.15 (0.14–0.15)	0.838
GIR	8.31 (7.74–8.93)	8.57 (7.79–9.42)	8.03 (7.20–8.95)	0.373
TyG	4.41 (4.38–4.43)	4.42 (4.38–4.46)	4.39 (4.35–4.42)	0.189
TG/HDL-C ratio	1.45 (1.36–1.55)	1.53 (1.39–1.68)	1.37 (1.27–1.47)	0.064

DM, diabetes mellitus; TSH, thyrotropin; fT4, free thyroxine; BMI, body mass index; FBG, Fasting blood glucose; TG, triglyceride; HDL-C, high-density lipoprotein cholesterol; HOMA-IR, homeostatic model assessment for insulin resistance; QUICKI, quantitative insulin-sensitivity check index; GIR, glucose to insulin ratio; TyG, triglyceride–glucose index; TG/HDL-C ratio, ratio of triglyceride to high-density lipoprotein cholesterol. Values represent weighted means and 95% confidence intervals for continuous variables and frequencies and weighted percentages for categorical variables.

**Table 2:** Association between thyroid function and insulin resistance indices.

	Independent Variables	Outcomes	Unadjusted Model			Adjusted Model ^a^		
			*β*	95% CI	*p* Value	*β*	95% CI	*p*
Boys	log HOMA-IR	TSH	−0.21	(−0.64, 0.22)	0.337	−0.17	(−0.65, 0.31)	0.488
	log HOMA-IR	fT4	−0.03	(−0.07, 0.00)	0.053	0.01	(−0.04, 0.05)	0.798
	TyG	TSH	0.41	(−0.18, 0.99)	0.172	0.53	(−0.05, 1.12)	0.075
	TyG	fT4	0.04	(−0.03, 0.11)	0.254	0.05	(−0.02, 0.11)	0.173
	log TG/HDL-C	TSH	0.09	(−0.17, 0.36)	0.482	0.14	(−0.12, 0.41)	0.288
	log TG/HDL-C	fT4	0.02	(−0.02, 0.05)	0.321	0.02	(−0.01, 0.05)	0.237
	QUICKI	TSH	10.08	(−10.83, 31.00)	0.343	7.88	(−15.72, 31.48)	0.511
	QUICKI	fT4	1.66	(0.02, 3.30)	0.047	−0.12	(−2.18, 1.93)	0.908
	log GIR	TSH	0.27	(−0.19, 0.73)	0.244	0.26	(−0.24, 0.75)	0.311
	log GIR	fT4	0.04	(0.00, 0.08)	0.049	0.00	(−0.05, 0.05)	0.928
Girls	log HOMA-IR	TSH	−0.25	(−0.73, 0.23)	0.300	−0.30	(−0.81, 0.20)	0.232
	log HOMA-IR	fT4	−0.04	(−0.10, 0.01)	0.141	−0.05	(−0.11, 0.00)	0.045
	TyG	TSH	0.50	(−0.28, 1.28)	0.210	0.49	(−0.36, 1.34)	0.261
	TyG	fT4	−0.05	(−0.15, 0.05)	0.327	−0.07	(−0.17, 0.03)	0.173
	log TG/HDL-C	TSH	0.17	(−0.16, 0.51)	0.310	0.17	(−0.20, 0.54)	0.362
	log TG/HDL-C	fT4	−0.02	(−0.07, 0.02)	0.276	−0.03	(−0.07, 0.01)	0.103
	QUICKI	TSH	12.42	(−11.35, 36.19)	0.304	15.86	(−9.31, 41.04)	0.215
	QUICKI	fT4	1.78	(−0.93, 4.48)	0.197	2.30	(−0.26, 4.87)	0.078
	log GIR	TSH	0.20	(−0.28, 0.68)	0.416	0.24	(−0.27, 0.75)	0.347
	log GIR	fT4	0.05	(−0.01, 0.11)	0.082	0.06	(0.01, 0.12)	0.022

TSH, thyrotropin; fT4, free thyroxine; HOMA-IR, homeostatic model assessment for insulin resistance; TyG, triglyceride–glucose index; TG/HDL-C ratio, ratio of triglyceride to high-density lipoprotein cholesterol; QUICKI, quantitative insulin-sensitivity check index; GIR, glucose to insulin ratio. ^a^ Adjusted for sex, age, household income (quartiles, continuous), BMI, muscle exercise (yes/no), and parental history of DM (yes/no).

**Table 3:** Association between thyroid function and insulin resistance based on the presence or absence of obesity.

			Normal Weight			Overweight & Obesity		
	Independent Variables	Outcomes	*β*	95% CI	*p*	*β*	95% CI	*p*
Boys	log HOMA-IR	TSH	−0.17	(−0.69, 0.36)	0.534	−0.15	(−1.16, 0.86)	0.770
	log HOMA-IR	fT4	−0.01	(−0.06, 0.04)	0.773	0.08	(0.00, 0.15)	0.037
	TyG	TSH	0.24	(−0.52, 1.00)	0.537	1.01	(0.07, 1.95)	0.035
	TyG	fT4	0.05	(−0.03, 0.13)	0.204	0.03	(−0.08, 0.13)	0.617
	log TG/HDL-C	TSH	−0.04	(−0.36, 0.28)	0.804	0.42	(0.00, 0.84)	0.051
	log TG/HDL-C	fT4	0.02	(−0.02, 0.06)	0.287	0.01	(−0.04, 0.05)	0.753
	QUICKI	TSH	7.12	(−18.66, 32.89)	0.586	7.18	(−49.19, 63.56)	0.801
	QUICKI	fT4	0.40	(−1.90, 2.70)	0.732	−4.14	(−7.97, −0.31)	0.034
	log GIR	TSH	0.28	(−0.22, 0.78)	0.267	0.22	(−0.93, 1.38)	0.704
	log GIR	fT4	0.01	(−0.05, 0.06)	0.812	−0.07	(−0.16, 0.01)	0.092
Girls	log HOMA-IR	TSH	−0.29	(−0.99, 0.40)	0.408	−0.15	(−0.66, 0.36)	0.573
	log HOMA-IR	fT4	−0.01	(−0.08, 0.06)	0.736	−0.12	(−0.19, −0.05)	0.002
	TyG	TSH	0.13	(−0.96, 1.22)	0.815	1.38	(0.32, 2.44)	0.011
	TyG	fT4	−0.05	(−0.18, 0.07)	0.408	−0.12	(−0.25, 0.01)	0.079
	log TG/HDL-C	TSH	0.03	(−0.45, 0.52)	0.894	0.44	(−0.05, 0.93)	0.081
	log TG/HDL-C	fT4	−0.03	(−0.08, 0.02)	0.305	−0.06	(−0.11, 0.00)	0.046
	QUICKI	TSH	15.12	(−18.08, 48.32)	0.369	7.68	(−17.00, 32.36)	0.540
	QUICKI	fT4	0.44	(−2.85, 3.74)	0.791	6.60	(2.46, 10.73)	0.002
	log GIR	TSH	0.14	(−0.63, 0.91)	0.719	0.39	(−0.07, 0.84)	0.095
	log GIR	fT4	0.03	(−0.05, 0.11)	0.498	0.14	(0.06, 0.22)	0.001

TSH, thyrotropin; fT4, free thyroxine; HOMA-IR, homeostatic model assessment for insulin resistance; TyG, triglyceride–glucose index; TG/HDL-C ratio, ratio of triglyceride to high-density lipoprotein cholesterol; QUICKI, quantitative insulin-sensitivity check index; GIR, glucose to insulin ratio. Beta coefficients were obtained by adjusting for age, household income (quartiles, continuous), BMI, muscle exercise (yes/no), and parental history of DM (yes/no).

## Data Availability

Data supporting the reported results can be found via publicly available datasets (https://knhanes.kdca.go.kr/knhanes/sub03/sub03_02_05.do (accessed on 26 January 2024)).

## Supplementary Materials

The following supporting information can be downloaded at: https://www.mdpi.com/article/10.3390/children11030370/s1, Table S1: Association between basic characteristics and thyroid function.
