# Supplementary material for: Association between Thyroid Function and Insulin Resistance Indices in Korean Adolescents: Findings from the 2014–2015 Korea National Health and Nutrition Examination Survey
Source: Children (Basel). 2024 Mar 20;11(3):370. doi: 10.3390/children11030370 (PMC10969090; doi:10.3390/children11030370)
Supplement: Supplementary file 1 [file children-11-00370-s001.zip › children-2894538-supplementary.pdf]

Supplementary Materials

Supplemental Table S1. Association between basic characteristics and thyroid function.

		TSH (uIU/mL)				fT4 (ng/dL)			
		β	lower 95% CI	upper 95% CI	*P*	*β*	lower 95% CI	upper 95% CI	*P*
Boys	Age (yrs)	-0.051	-0.153	0.052	0.333	0.016	0.006	0.026	0.002
	Household income (ordinal)	0.019	-0.182	0.219	0.855	-0.008	-0.029	0.012	0.430
	Aerobic exercise	0.230	-0.110	0.571	0.184	0.000	-0.055	0.055	0.997
	Muscle exercise	0.077	-0.299	0.453	0.687	-0.032	-0.073	0.008	0.115
	Parental history of DM	-0.272	-0.847	0.303	0.353	-0.017	-0.123	0.089	0.754
	Waist circumference (cm)	0.002	-0.013	0.018	0.779	-0.001	-0.003	0.001	0.201
	BMI (kg/m^2)	-0.003	-0.045	0.039	0.875	-0.004	-0.008	0.001	0.131
Girls	Age (yrs)	-0.072	-0.170	0.025	0.143	-0.001	-0.011	0.010	0.901
	Household income (ordinal)	0.011	-0.167	0.189	0.902	0.008	-0.009	0.025	0.372
	Aerobic exercise	-0.170	-0.578	0.238	0.413	-0.009	-0.053	0.036	0.709
	Muscle exercise	-0.417	-0.860	0.026	0.065	-0.028	-0.084	0.029	0.335
	Parental history of DM	-0.061	-0.941	0.820	0.892	-0.010	-0.158	0.139	0.896
	Waist circumference (cm)	-0.008	-0.030	0.015	0.506	-0.001	-0.003	0.001	0.286
	BMI (kg/m^2)	-0.028	-0.074	0.017	0.224	-0.003	-0.008	0.002	0.193

DM, diabetes mellitus; TSH, thyrotropin; fT4, free thyroxine; BMI, body mass index.
